# Comparison Between the Treatment Modalities for Graves' Disease at King Abdulaziz Medical City, Jeddah

**DOI:** 10.7759/cureus.6730

**Published:** 2020-01-21

**Authors:** Hawazen A Zarif, Sultan S Alam, Abdulrahman Baashar, Abdulaziz Alsharif, Mashael Alhilabi

**Affiliations:** 1 Medicine / Endocrinology, Ministry of the National Guard - Health Affairs, King Abdullah International Medical Research Center, Jeddah, SAU; 2 Internal Medicine, King Saud Bin Abdulaziz University for Health Sciences / King Abdulaziz Medical City, Ministry of National Guard Health Affairs, Jeddah, SAU; 3 Internal Medicine, King Saud Bin Abdulaziz University for Health Sciences, Jeddah, SAU; 4 Internal Medicine, King Saud Bin Abdulaziz University for Health Sciences / King Abdullah International Medical Research Center / King Abdulaziz Medical City, Jeddah, SAU; 5 Internal Medicine / Endocrinology, King Abdulaziz Medical City / King Abdullah International Medical Research Center, Jeddah, SAU

**Keywords:** graves' disease, thyrotoxicosis, hyperthyroidism, antithyroid medications, antithyroid drugs, atd, radioactive iodine ablation, rai, treatment modalities, treatment choices

## Abstract

Background

Graves’ disease is the most common cause of thyrotoxicosis. It can be treated using three different modalities, which include anti-thyroid drugs (ATD), radioactive iodine (RAI), and near-total thyroidectomy. This cohort study aimed to assess the treatment modality preferred at King Abdulaziz Medical City (KAMC) and to compare the treatment options in relation to the prognosis of the disease.

Methods

A retrospective cohort study was conducted on a total of 100 patients with Graves’ disease who were treated and followed up in the endocrine clinics at KAMC between January 2013 and December 2018. Data on age at diagnosis, duration of illness, treatment modality, and response to treatment were extracted from paper and electronic medical files and analyzed.

Results

A total of 100 patients with Graves’ disease were included in this cohort study. The ratio of female:male was 2:1. The median age in years was 32 (16). They were treated with ATD (60%), RAI ablation (40%), and none were treated by surgery. The remission rate was 53.3% for patients treated with ATD and 95% for RAI ablation. Hypothyroidism occurred in 90% of the responders to RAI and in 12% that were treated with ATD. Most of the patients that relapsed underwent RAI as the second line of treatment. Their remission rate was 78.6%.

Conclusion

ATD was the treatment modality mostly used for Graves’ disease in our center. It resulted in a remission rate of 53%, which is higher than reported in national studies. Although the rate of remission post RAI ablation was as high as 95%, most patients developed hypothyroidism.

## Introduction

Hyperthyroidism (thyrotoxicosis) is a state of overproduction of thyroid hormone that can result in clinical manifestations ranging from mild symptoms, such as tremors, anxiety, and palpitations, to severe symptoms associated with morbidity and mortality. Graves’ disease is the most common cause, as 76% of all causes of thyrotoxicosis is related to this condition [1]. It is an autoimmune disease caused by circulating antibodies known as thyroid-receptor antibodies (TRab) [1]. When thyrotropin receptors are stimulated by TRab, the gland starts to grow and the follicles excessively synthesize thyroid hormone. Though Graves’ disease can occur in any age group, it commonly occurs in women aged 30 to 50 years [1]. It is treated using three different modalities: antithyroid drugs (ATD), radioactive iodine (RAI) ablation, or surgery [2]. ATD, mainly methimazole, carbimazole, and propylthiouracil (PTU), block the synthesis of thyroid hormones [1]. A cross-sectional study done to evaluate the remission rate of ATD in patients with Graves’ disease showed that 41.9% of patients who took ATD during the follow-up period of 18 months went into remission. For those who do not respond to treatment in 12-18 months, an alternative treatment should be considered [3]. RAI ablation is the most widely used therapeutic option in the United States for the treatment of Graves’ disease [4]. Among 261 patients, 86% developed hypothyroidism or were euthyroid post RAI ablation and 14% had persistent hyperthyroidism and were given a second dose of RAI [4]. Similarly, results from a study in China found that 49.7% of patients became euthyroid, 38.3% became hypothyroid, and 12.0% remained hyperthyroid post RAI treatment [5]. Although surgery offers rapid control of hyperthyroidism, it is usually reserved for patients with a recurrence of Graves’ disease after treatment, when the goiter is large, or when other modalities are contraindicated [2].

There is a wide variation in treatment preferences among physicians. A study done by the American Thyroid Association (ATA), the European Thyroid Association (ETA), and the Japan Thyroid Association (JTA) showed that radioiodine was the therapy of choice for ATA respondents (69%), followed by ETA (22%), and then JTA respondents (11%). However, JTA respondents (88%) preferred anti-thyroid drugs as first-line therapy followed by ETA (77%) and then ATA (30.5%) [6].

 Little is known about the treatment preferences and outcomes of patients with Graves’ disease among the Saudi population. Therefore, this study aims to assess the treatment modality preferred at King Abdulaziz Medical City (KAMC), in Jeddah, Saudi Arabia for the treatment of Graves’ disease and to compare between the three treatment options available in relation to the prognosis of the disease.

## Materials and methods

KAMC is a tertiary care center that provides medical care services to Saudi citizens. All consecutive patients diagnosed to have Graves’ disease who were seen and followed in the endocrine clinics at KAMC between January 2013 and December 2018 were eligible. Patients older than 18 and less than 65 years at the time of diagnosis were included. The patients were required to have been treated with ATD, RAI, or surgery. The exclusion criteria were other causes of hyperthyroidism such as toxic multinodular goiter, solitary toxic nodule, and thyroiditis. In addition, patients who used medications that can cause hyperthyroidism such as amiodarone or alpha interferon were excluded. The sample size was calculated using alpha 0.05, power 80%, and effect size (the difference in the proportion of remission between RAI and ATD) = 0.30. The sample size was estimated to be 88. We used the non-probability consecutive sampling technique. Data were extracted from paper and electronic medical files and collected on a data collection sheet. The variables were gender, age at diagnosis, duration of illness, type of treatment, and response to treatment. We calculated the proportion of patients receiving each modality of treatment. We determined the first-line treatment most widely used by the endocrinologists in our hospital and patient characteristics and measured the remission rate for each modality. Statistical analysis was performed using IBM SPSS Statistics (IBM Corp, Armonk, NY) software package. For descriptive statistics, we used counts and proportions for categorical variables. After being tested for normality, continuous data were not symmetrically distributed and were reported as medians and interquartile range. The chi-square and Fisher's exact test were used to compare categorical data and the Mann-Whitney test was used for continuous data. A p-value of less than 0.05 was considered significant.

The study was approved by the Institutional Review Board of King Abdullah Medical Research Centre. The analysis performed was retrospective and no patient consent was needed.

## Results

A total of 100 patients with Graves’ disease were included in this study, which compares the outcome of three modalities: ATD, RAI, and surgery. Among those included, 33 were males and 67 were females. The median age and interquartile range (IQR) in years was 32 (16). As a first-line treatment, 60% (18 males and 42 females) were treated with ATD; the remaining 40% (15 males and 25 females) underwent RAI ablation. None of those patients had surgery for the treatment of Graves’ disease (Table 1). Among the 60 who were treated with ATD, 53% had a remission, whereas, among the 40 patients that were treated with RAI ablation, 95% had remission (P < 0.001) (Table 2). For those who underwent RAI as the first line of treatment, 90% of the patients became hypothyroid. On the other hand, in those treated by ATD, 12% developed hypothyroidism post-treatment (P < 0.001). Data were available for only 15 patients that failed first-line therapy and were treated by second-line therapy. The majority (14; 93.3%) underwent RAI ablation as the second line. Among them, 13 patients have failed ATD as the first line and one failed RAI ablation. The remission rate was 78.6%. Only one patient (6.7) was treated by ATD after the failure of RAI. This patient relapsed after completion of a course of ATD.

**Table 1:** Baseline characteristics of the subjects included in the study N=number of subjects; Sample size=100; Number of subjects = percentages RAI, Radioactive Iodine Ablation; IQR, Interquartile Range

Patients characteristics	Descriptive statistics
Gender, N	
Male	33
Female	67
Age of diagnosis, median (IQR) years	32 (16)
Drugs as the first line, N	60
RAI as the first line, N	40

**Table 2:** Comparison between the treatment modalities N=number of subjects RAI, Radioactive Iodine Ablation

Modality of treatment	Remission N (%)	P-value	Hypothyroidism N (%)	P-value
Drugs	32 (53)	<0.001	7 (12)	<0.001
RAI	38 (95)		36 (90)	

## Discussion

Our study included 100 patients treated and followed up at KAMC for Graves’ disease. Most of the patients were female (ratio 2:1) and the median age was 32. A similar study was conducted in King Khalid University (KKU), Riyadh, and included 194 Graves’ disease patients; the ratio of the females was higher 2.9:1 and the mean age was 32 ± 0.9 years [7]. In King Abdulaziz University (KAU), Jeddah, out of a total of 203 patients, the ratio of females to males was 3.8:1 and the mean age was 35.49 ± 10.86 years [8]. They included patients with different etiologies for hyperthyroidism. Graves' disease was the most common (69%), followed by toxic multinodular goiter (29%) and other causes (2%).

Treatment preferences vary depending on the center. In our center, 60% were treated with ATD, mainly methimazole (MMI), 40% underwent RAI ablation, and none underwent subtotal thyroidectomy. The remission rate was 53% using ATD and 95% using RAI ablation. Among the patients treated with RAI, 90% became hypothyroid while 12% treated with ATD developed hypothyroidism.

In comparison to other local studies, in KKU, 38% were treated with ATD, 49% of patients were treated with RAI, and 13% underwent subtotal thyroidectomy [8]. The remission rate was 26% on ATD and 83% on RAI ablation [9]. In a study conducted at Al-Nour Hospital in Makkah, a total of 50 patients were followed up after five years of treatment, 74% were treated with ATD, 16% with RAI ablation, and 8% had surgery (Abstract presented at the 22nd Joint Meeting of the British Endocrine Societies; Karawagh A, Abdelaziz M: Outcome of carbimazole, radioactive iodine and subtotal thyroidectomy in the treatment of Graves. 2003; https://www.endocrine-abstracts.org/ea/0005/ea0005p279). The remission rate on ATD was 19%, RAI 88%, and surgery 75%. Hypothyroidism occurred in 37% of patients given RAI, 50% were treated with surgery, and 5% of patients were treated with ATD. In KAU, 72.68% of patients were treated with radioactive iodine and 23% received ATD. Thirty-seven percent developed hypothyroidism post RAI [8]. At King Faisal Specialist Hospital and Research Center in Riyadh, 78% were treated with RAI ablation, 73% became hypothyroid during the observation period of three months to eight years (median, 1.5 years) [10].

In comparison to international studies, we compared our results to a cohort study that included 720 adults diagnosed with Graves' disease from January 2002 to December 2008 conducted at the Mayo Clinic [11]. The most used therapy was RAI 75.4%, followed by ATD 16.4% and then thyroidectomy 2.6%. The remission rate of drugs was 51.7% compared with 92% for RAI while surgery had a 100% success rate. Although RAI was more popular in this cohort, the remission rates reported for both ATD and RAI were very similar to our study.

Data were available from 15 patients that failed first-line treatment, 13 (86.7) failed medical therapy, and 1 (6.7) failed RAI. They were treated by RAI as the second-line therapy. The remission rate was 78.6% after RAI. The only patient that received medications after RAI relapsed after ATD was discontinued. None of our patients received another course of antithyroid medications after the failure of the first course. Prolonged low doses of MMI may be an option for relapsed Graves' disease patients, particularly for patients that had Graves' ophthalmopathy or for those who refuse RAI ablation. In a non-randomized retrospective analysis of relapsed Graves' disease previously treated by ATD for one to two years, patients were treated with RAI treatment and L-thyroxine replacement or a low dose of MMI. Thyroid dysfunction, Graves’ ophthalmopathy deterioration, and weight gain were higher in the RAI group. They concluded that low doses of MMI are safe and effective and have better outcomes than RAI treatment [12].

## Conclusions

ATD was the treatment modality mostly used for Graves’ disease in our center. It resulted in a remission rate of 53%, which is higher than national studies and comparable to the results from the Mayo Clinic. The rate of remission post RAI ablation was as high as 95% and that was comparable with local and international studies. However, the rate of hypothyroidism post RAI was much higher in comparison to both national and international studies. The remission rate for patients that failed first-line treatment and received RAI ablation as a second line was 78.6%.
